# Satisfactory immediate spontaneous correction may not mean satisfactory final results for moderate TL/L curves after selective thoracic fusion in AIS patients

**DOI:** 10.1186/s12891-023-06591-8

**Published:** 2023-07-01

**Authors:** Yanbin Zhang, Jing Bai, Bin Xiao, Jianguo Zhang, Da He, Yonggang Xing, Bo Liu

**Affiliations:** 1 Department of Spine Surgery, Beijing Jishuitan Hospital, Xicheng District Xinjiekou No. 31 East Street, Beijing, 100035 P.R. China; 2 Department of Trauma and Joint, The Third Affiliated Hospital of Beijing University of Traditional Chinese Medicine, Chaoyang District Anwai Xiaoguan Street No. 51, Beijing, 100029 P.R. China; 3 Department of Orthopedics of Peking Union Medical College Hospital, 1Shuai Fu Yuan, Beijing, 100730 P.R. China

**Keywords:** Adolescent idiopathic scoliosis, Selective thoracic fusion, Risk factor, Correction loss, Mismatch

## Abstract

**Background:**

Few studies have focused on the chronic spontaneous behavior of the unfused TL/L curve during follow-up. The purpose of the present study was to explore the behavior of the unfused TL/L curve during a long-term follow-up to identify the risk factors for correction loss.

**Methods:**

Sixty-four age-matched female AIS patients undergoing selective thoracic fusion were enrolled. Patients were divided into 2 groups according to whether there was correction loss. Risk factors for correction loss of the unfused TL/L curves were analyzed. The relationship and difference between the immediate postoperative thoracic and TL/L Cobb angles were explored.

**Results:**

The TL/L Cobb angle was 28.17° before surgery, 8.60° after surgery, and 10.74° at the final follow-up, with a correction loss of 2.14°. Each subgroup contained 32 cases. A smaller postoperative TL/L Cobb angle was the only risk factor that was independently associated with TL/L correction loss. In the LOSS group, there was a significant difference and no correlation between the immediate postoperative TL/L and the thoracic Cobb angle. In the NO-LOSS group, there was a moderate correlation and no difference between them.

**Conclusion:**

A smaller immediate postoperative TL/L Cobb angle may have been associated with TL/L correction loss during the long-term follow-up. Thus, good immediate postoperative spontaneous correction may not mean a satisfactory outcome at the final follow-up after STF. Mismatch between thoracic and TL/L Cobb angles immediately after surgery may also be related to correction loss of the unfused TL/L curves. Close attention should be paid in case of deterioration.

**Supplementary Information:**

The online version contains supplementary material available at 10.1186/s12891-023-06591-8.

## Background

Adolescent idiopathic scoliosis (AIS) is a three-dimensional (3D) deformity of the spine that predominantly affects individuals aged 10 to 17. Fusion level selection in the surgical treatment of AIS patients with structural major thoracic (MT) and secondary thoracolumbar or lumbar (TL/L) curves remains a great challenge [1–4].

In nonselective fusion, instrumentation of both curves sacrifices the mobile segments of the spine. But in selective thoracic fusion (STF), progression of the uninstrumented lumbar curve or coronal imbalance may occur [5]. STF dates back to the era of Harrington instrumentation with the purpose of sparing lumbar motion [4]. At present, pedicle screws are predominantly used because of their powerful corrective force [6].

Various studies have focused on the prognosis or prediction of the unfused lumbar curve after STF [2, 5, 7, 8] and the change from preoperation to the final follow-up. However, few studies have focused on the chronic spontaneous behavior of the unfused TL/L curve during follow-up (the change from immediate postoperation to the final follow-up) or the risk factors for its correction loss. In selective TL/L fusion, our previous study showed that higher flexibility and better immediate correction were risk factors for correction loss of the unfused thoracic curve during the follow-up [9]. Therefore, the purposes of the present study were to explore the behavior of the unfused TL/L curve after STF during the two-year follow-up and to identify the risk factors for its correction loss.

Our specific goals were to (1) evaluate the radiographic outcome of STF, (2) compare the difference between two age- and sex-matched subgroups, (3) identify the risk factors for correction loss of the unfused lumbar curve, and (4) explore the influence of immediate postoperative mismatch between thoracic and TL/L curves.

## Materials and methods

### Patient selection

After the institutional review board (IRB) approved the study, patients with Lenke 1 AIS were identified retrospectively. The Lenke classification [3] criteria were utilized and confirmed with another independent surgeon. It was considered selective fusion for AIS patients with MT and secondary TL/L curves if the TL/L curves were unfused. The inclusion criteria were as follows: patients diagnosed with Lenke 1 AIS with a minimal follow-up of 2 years; underwent posterior STF. The exclusion criteria were as follows: incomplete data or poor radiographic images that do not allow measurement; age and sex were not matched between subgroups according to Subgroup Analysis section.

### Surgical technique

During preoperative planning, the last substantially touched vertebra (LSTV) [10] was selected as the lower instrumented vertebra (LIV). The patient was placed prone on a radiolucent spinal frame after general anesthesia. After surgical exposure, the pedicle screws were placed and the posterior elements were released if necessary. Then the rods were placed. The curve was corrected with direct apical vertebra rotation, rod rotation and compression and/or distraction. Then, the bone graft was applied. Intraoperative neurophysiological monitoring was used.

### Radiographic measurements

Radiographic measurements were performed on the Surgimap (Nemaris) by 2 independent staff members on standing whole-spine posteroanterior and lateral radiographs taken before surgery, 1 month after surgery, and at the most recent follow-up. Postoperative X-rays were taken 2 weeks after surgery, instead of at the first erect instance, to rule out the influence of postoperative pain and to allow the patients to recover their physiological balance [11]. Before surgery, supine side-bending films were also taken. Coronal parameters included MT and its convex side-bending Cobb angle, TL/L and its convex side-bending Cobb angle, lower instrumented vertebra tilt (LIV Tilt), global coronal balance and apical vertebral translation (AVT) as previously described [9]. Sagittal alignments included global sagittal balance or sagittal vertical axis, thoracic kyphosis, lumbar lordosis, and thoracolumbar junction. The correction rate was defined as (preoperative Cobb angle – immediate postoperative or final Cobb angle)/preoperative Cobb angle. The correction loss was defined as the final Cobb angle – immediate postoperative Cobb angle. The Cincinnati correction index was calculated as the immediate postoperative correction rate/preoperative flexibility [6].

### Subgroup Analysis

According to TL/L correction loss, all cases were divided into 2 age- and sex-matched subgroups. If the TL/L Cobb angle improved or was maintained during the follow-up with a negative or no correction loss, the case was allocated to the NO-LOSS group. If the TL/L Cobb angle deteriorated with a positive correction loss, the case belonged to the LOSS group. Comparison and correlation analyses were performed to explore the difference between these two subgroups and the risk factors for correction loss of the unfused TL/L curve.

### Statistical analysis

We presented summary statistics by means and standard deviations (SDs) for continuous variables and frequencies for categorical variables. Paired or independent t tests were used for continuous variables obeying a normal distribution. Nonparametric tests were utilized if the data did not obey a normal distribution. A multivariate binary logistic regression model with forward stepwise elimination (Conditional) was created to evaluate the adjusted association of each potential risk factor predicting correction loss of the unfused TL/L curves. We considered variables with a univariate significance level of less than 0.05 for inclusion in the multivariate analysis. For regression models, the adjusted odds ratio and their subsequent 95% confidence interval (CI) were reported. Pearson correlation was employed to examine the relationship between immediate postoperative MT and TL/L Cobb angles. The strength of the correlation was defined by the r value: negligible correlation (r < 0.3), weak correlation (0.3 < r < 0.5), moderate correlation (0.5 < r < 0.7), strong correlation (0.7 < r < 0.9) and very strong correlation (r > 0.9). We performed all analyses using SPSS (version 23.0, IBM Corp., USA). A p value < 0.05 was considered significant.

## Results

### General Information

We identified 73 cases of AIS in our database, and 9 patients were excluded because they were unmatched for age and sex. Finally, 64 patients were age-matched female patients with an average age of 14.3 years old (range, 11–19 years). The follow-up duration averaged 36.9 months (range, 24–61 months). (Table 1)

**Table 1:** Demographic Details of the Patients

Parameters	Value
Total Patients	64
Sex	Female
Age (years)	14.3 ± 2.2 (11–19)
BMI (kg/m2)	17.13 ± 2.12 (14.14–23.41)
Lumbar Modifier	
A	21
B	43
Risser Sign	
0	7
1	2
2	12
3	13
4	19
5	11

### Surgical Outcomes

General coronal and sagittal measurements are shown in Table 2. Only 1 patient in the LOSS group underwent revision surgery to fuse the progressive TL/L curve. The TL/L Cobb angle was 28.17 ± 5.99° before surgery and 8.60 ± 6.28° immediately after surgery (p < 0.001). At the final follow-up, it had deteriorated significantly to 10.74 ± 5.34° (p: 0.045), with a correction loss of 2.14 ± 6.71°.

**Table 2:** Comparison of Coronal and Sagittal Parameters

Parameters	Pre-op	Post-op	Follow-up	p Value		
				Pre-op vs. Post-op	Pre-op vs. Follow-up	Post-op vs. Follow-up
MT						
Cobb(°)	45.74 ± 6.93	9.98 ± 7.52	13.64 ± 7.75	＜0.001^*^	＜0.001^*^	＜0.001^*^
Bending Cobb(°)	21.33 ± 9.47					
Flexibility(%)	54.22 ± 16.96					
Immediate Correction(%)		78.82 ± 14.93				
LIV Tilt(°)	19.8 ± 6.2	5.7 ± 3.6	6.7 ± 4.1	＜0.001^*^	＜0.001^*^	0.032^*^
TL/L						
Cobb(°)	28.17 ± 5.99	8.60 ± 6.28	10.74 ± 5.34	＜0.001^*^	＜0.001^*^	0.045^*^
Bending Cobb(°)	-2.12 ± 9.21					
Flexibility(%)	106.88 ± 29.43					
Immediate Spontaneous Correction(%)		73.90 ± 17.65				
Correction Loss(°)			2.14 ± 6.71			
Coronal Balance(mm)	11.61 ± 9.07	15.47 ± 13.48	7.68 ± 10.94	0.122	0.087	0.001^*^
T5-12 Kyphosis(°)	25.74 ± 9.34	21.93 ± 5.34	22.29 ± 5.39	0.004^*^	0.007^*^	0.283
T10-L2 Alignment(°)	-0.12 ± 6.02	-0.24 ± 4.50	0.45 ± 2.71	0.916	0.589	0.136
L1-S1 Lordosis(°)	31.24 ± 11.94	31.50 ± 9.76	30.38 ± 9.10	0.75	0.435	0.039^*^
SVA(mm)	5.62 ± 13.03	11.21 ± 14.54	3.71 ± 9.08	0.001^*^	0.223	0.002^*^

TM: major thoracic curve, LIV Tilt: lower instrumented vertebra tilt, TL/L: thoracolumbar or lumbar curve, SVA: sagittal vertical axis, * means significant difference

### Risk factors for correction loss

TL/L curves did not deteriorate after spontaneous correction in 32 cases in the NO-LOSS group, while deteriorated in 32 cases in the LOSS group. The correction losses were − 3.43 ± 3.91° (range − 14°-0°) and 7.71 ± 3.42° (range 2°-14°), respectively. Comparisons were made using the univariate analysis (Table 3). General conditions (including age, Risser signs and follow-up duration) and preoperative Cobb angles, especially the TL/L Cobb angle, its convex side-bending Cobb angle and flexibility, were not significantly different. After surgery, patients in the LOSS group had a smaller immediate postoperative MT Cobb angle (p: 0.044), smaller immediate postoperative TL/L Cobb angle (p: 0.008), higher TL/L immediate spontaneous correction rate (p: 0.014), and higher immediate postoperative coronal balance (p: 0.027) than those in the NO-LOSS group. However, after a long-term follow-up, the patients in the LOSS group had a larger TL/L Cobb angle (p＜0.001), but the MT Cobb angle was not significantly different (p: 0.155).

**Table 3:** Univariate Analysis of Risk Factors for Correction Loss of TL/L curves

Parameters	Correction Loss		p Value
	LOSS	NO-LOSS	
Age(years)	14.29 ± 2.26	14.28 ± 2.22	0.993
Risser Signs	3 ± 1.45	3.05 ± 1.32	0.912
Follow-up Duration	40.6 ± 13.0	35.8 ± 9.8	0.179
MT Cobb(°)			
Pre-op	44.71 ± 4.75	46.76 ± 8.58	0.184
Bending Cobb	18.76 ± 8.26	23.90 ± 10.08	0.508
Flexibility(%)	58.91 ± 15.35	49.54 ± 17.54	0.768
Post-op	8.43 ± 6.15	11.52 ± 8.55	0.044^*^
Immediate Correction(%)	81.36 ± 13.51	76.28 ± 16.15	0.196
Follow-up	13.00 ± 7.18	14.29 ± 8.40	0.155
LIV Tilt(°)			
Pre-op	20.4 ± 6.3	19.2 ± 5.9	0.513
Post-op	6.3 ± 4.4	5.1 ± 3.7	0.149
TL/L Cobb(°)			
Pre-op	27.57 ± 6.33	30.10 ± 5.19	0.501
Bending Cobb	-5.07 ± 8.47	0.83 ± 9.16	0.602
Flexibility	117.50 ± 29.77	96.27 ± 25.56	0.25
Post-op	4.38 ± 3.03	12.81 ± 5.87	0.008^*^
Immediate Spontaneous Correction (%)	85.66 ± 9.32	62.14 ± 16.16	0.014^*^
Follow-up	12.10 ± 3.33	9.38 ± 6.59	＜0.001^*^
Cobb Ratio MT:TL/L	1.58 ± 0.32	1.44 ± 0.34	0.807
Cincinnati Correction Index	77.41 ± 21.63	67.15 ± 22.96	0.909
MT AVT(mm)			
Pre-op	35.19 ± 9.12	36.95 ± 10.59	0.513
TL/L AVT(mm)			
Pre-op	12.24 ± 6.01	15.05 ± 5.75	0.955
AVT Ratio MT:TL/L	3.98 ± 3.42	3.13 ± 2.27	0.35
Coronal Balance(mm)			
Pre-op	10.71 ± 8.09	11.70 ± 9.91	0.188
Post-op	17.30 ± 15.44	13.44 ± 11.00	0.027^*^
Follow-up	8.42 ± 12.85	7.13 ± 9.69	0.536
T5-12 Kyphosis(°)			
Pre-op	25.62 ± 9.65	25.86 ± 9.26	0.935
Post-op	21.76 ± 5.23	22.10 ± 5.57	0.843
T10-L2 Alignment(°)			
Pre-op	-0.24 ± 6.74	0 ± 5.38	0.9
Post-op	0.29 ± 4.86	-0.76 ± 4.17	0.458
L1-S1 Lordosis(°)			
Pre-op	32.24 ± 11.85	30.24 ± 12.23	0.593
Post-op	31.95 ± 10.15	31.05 ± 9.58	0.768
SVA(mm)			
Pre-op	5.71 ± 13.63	5.52 ± 12.74	0.963
Post-op	10.19 ± 17.22	12.24 ± 11.59	0.654

TM: major thoracic curve, TL/L: thoracolumbar or lumbar curve, AVT: apical vertebral translation, SVA: sagittal vertical axis, * means significant difference

**Fig. 1:**
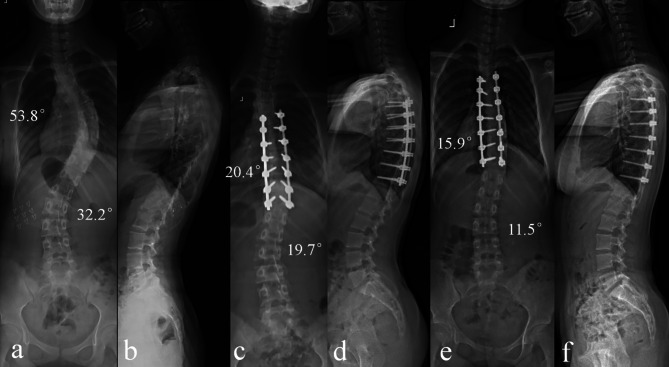
A typical case in group NO-LOSS: a 13-year-old female AIS patient underwent posterior selective thoracic fusion. The TL/L Cobb angle was 32.2° before surgery (a-b) and was corrected to 19.7° (c-d). After a follow-up of 46 months, the TL/L Cobb angle was 11.5°, with an improvement of 8.2° (e-f)

**Fig. 2:**
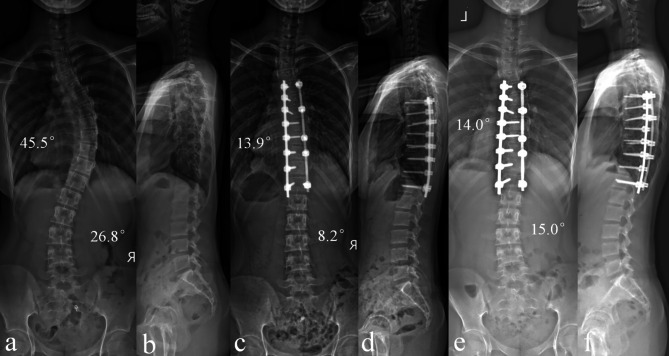
A typical case in the LOSS group: A 13-year-old female AIS patient underwent posterior selective thoracic fusion. The TL/L Cobb angle was 26.8° before surgery (a-b) and was corrected to 8.2° (c-d). After a follow-up of 32 months, the TL/L Cobb angle was 15.0°, with a correction loss of 6.8° (e-f)

In the multivariate analysis, a smaller TL/L postoperative Cobb angle was the only risk factor that was independently associated with TL/L correction loss (odds ratio = 1.417; 95% CI: 1.160–1.731; p＜0.001) (shown in Table 4). Typical cases are shown in Figs. 1 and 2.

**Table 4:** Multivariate Analysis of Risk Factors for Correction Loss of TL/L curves

Parameter	Odds Ratio (95% CI)	95% CI		p
		Upper	Lower	
MT Cobb(°)				
Post-op				0.149
TL/L Cobb(°)				
Pre-op				0.314
Flexibility				0.295
Post-op	1.417	1.160	1.731	＜0.001*
Immediate Correction Rate(%)				0.68
Coronal Balance(mm)				
Post-op				0.072

MT: major thoracic curve, TL/L: thoracolumbar or lumbar curve, * means significant difference

### Mismatch between MT and TL/L Curves

Furthermore, we explored the relationship and difference between the immediate postoperative TL/L and MT Cobb angles. In the total group, the TL/L Cobb angle had a weak correlation with the MT Cobb angle (p: 0.023) and was not significantly different from the MT Cobb angle (p: 0.230). In the LOSS group, the TL/L Cobb angle had no correlation with the MT Cobb angle (p = 0.749) and was significantly different from the MT Cobb angle (p = 0.011). However, in the NO-LOSS group, the TL/L Cobb angle had a moderate correlation with the MT Cobb angle (p = 0.008) and was not significantly different from the MT Cobb angle (p = 0.420). (Table 5)

**Table 5:** Comparison and Relationship between Post-op MT and TL/L Cobb Angle

Group	MT Cobb Post-op(°)	TL/L Cobb Post-op(°)	Pearson Correlation		Paired t Test	
			r	p value	t	p value
Total	9.98 ± 7.52	8.60 ± 6.28	0.350	0.023^*^	1.22	0.230
LOSS	8.43 ± 6.15	4.38 ± 3.03	0.074	0.749	2.792	0.011^*^
NO-LOSS	11.52 ± 8.55	12.81 ± 5.87	0.561	0.008^*^	-0.822	0.420

MT: major thoracic curve, TL/L: thoracolumbar or lumbar curve, * means significant difference

## Discussion

### Mechanism and indications

The theoretical basis of STF is that following correction of the MT curve, forces are transmitted to the lumbar spine, inducing spontaneous lumbar correction [12, 13]. It remains unknown which AIS patterns should receive STF. A thoracic:lumbar curve ratio of more than 1:2 is generally considered an indication [3, 14]. Lumbar curve magnitude/flexibility and coronal balance are also taken into consideration [15].

### Clinical outcomes

STF for AIS gained satisfactory outcomes with pedicle screw constructs. Gebrelul et al. [5] reported 102 AIS patients undergoing STF using all-screw constructs, and the average rate of spontaneous correction of the TL/L curve was 43% at the 2-year follow-up. Chen et al. [16] showed a spontaneous correction rate of more than 70% of the TL/L curves for Lenke 1 and 2 AIS patients. Similar results were reported over a wide range of studies [7, [8], [12], [14], 17–19]. In the present study, after STF, the TL/L curve was corrected from 28.17 ± 5.99° preoperatively to 8.60 ± 6.28° postoperatively and remained at 10.74 ± 5.34° at the final follow-up, which was comparable to previous studies.

### Characteristics of lumbar compensation

After STF, progression of the residual TL/L curve may not only exacerbate coronal imbalance or shoulder imbalance [20] but may also be associated with diminished patient self-image [21]. Therefore, the behavior of the unfused TL/L curve gained focus over years. Bachmann et al. [1] from the USA found that selective fusion had a limited ability to change the lower lumbar vertebral segments, including the lumbosacral takeoff angle (the angle between the central sacral vertical line and a best-fit line through the center of S1, L5, and L4). They explained that the limited correction of the lower lumbar segments made worsening of coronal balance more likely with selective fusion. Therefore, spontaneous correction occurred mainly at the upper part of the unfused lumbar curve. Similar results were noted by researchers in China. Chen et al. [16] found that when choosing L1 as the LIV, the distal unfused lumbar segments’ compensation tended to decrease from the proximal end to the distal end, suggesting that the L1/2 and L2/3 discs significantly contributed to this compensation. These two studies focused on the difference in compensation between the upper and lower lumbar segments, but neither identified the risk factors for correction loss during the long-term follow-up nor explored the relationship between thoracic and TL/L curve magnitude.

### Risk factors for lumbar curve progression

The primary focus was the prediction or prognosis of the unfused lumbar spine. A wide range of risk factors or predictors have been recognized. In 2011, the preoperative lumbar Cobb angle and lumbosacral takeoff angle were reported to be predictors of the 2-year postoperative lumbar Cobb angle, and a predictive formula was calculated [22]. Then, the formula was tested in 2019. [1] Koller et al. [23] found that the preoperative TL/L Cobb angle and preoperative convex-bending TL/L Cobb angle were significant predictors for the final TL/L Cobb angle. Mason et al. [24] also developed a formula including the preoperative TL/L Cobb angle, preoperative MT Cobb angle and its convex-bending Cobb angle. Most of the identified factors were preoperative, and most previous literature focused on the change from preoperation to the final follow-up. Few studies have focused on correction loss of the unfused TL/L curve during the long-term, from immediate postoperation to final follow-up. In the present study, we recognized four risk factors for correction loss of the unfused TL/L spine in the univariate analysis, including a smaller postoperative MT Cobb angle, a smaller postoperative TL/L Cobb angle, a higher postoperative spontaneous correction rate of the lumbar curve and a larger postoperative coronal balance. Furthermore, in the multivariate analysis, a smaller postoperative TL/L Cobb angle was identified as an independent risk factor for lumbar correction loss during follow-up (p < 0.001, odds ratio: 1.417, 95% confidence interval: 1.160–1.731). Therefore, a smaller immediate postoperative TL/L curve may be associated with correction loss of the unfused TL/L curve.

The potential explanation for the above result was similar to our report in selective TL/L fusion [9]: the preoperative TL/L Cobb angles were similar between the LOSS and NO-LOSS groups (p = 0.501), but the postoperative TL/L Cobb angle was significantly smaller in the LOSS group than in the NO-LOSS group (p = 0.008). Thus, a higher spontaneous correction rate in the LOSS group caused a larger change in curve magnitude. This may increase the tension of the concave soft tissues, which contained more fibrosis and fatty involution [25], and thus exacerbate the tendency toward curve progression during the follow-up. Additionally, the flexible unfused TL/L segments were susceptible to this tension. On the other hand, in the NO-LOSS group, a smaller spontaneous correction rate may have led to relatively low soft tissue tension on the concave side of the unfused TL/L curve, so there was a lower risk of progression. Another reason may be that a smaller postoperative TL/L Cobb angle contributes to the mismatch between the MT and TL/L Cobb angle, which may be related to correction loss, as we discussed below. These explanations were our assumptive interpretation, and further studies are needed for a detailed mechanism.

### Mismatch between MT and TL/L Curves

The correction of the TL/L curve was said to echo the correction of the thoracic curve after STF. Although some authors have reported that there is no relationship between the correction of the thoracic and TL/L curves after STF with the Harrington system and sublaminar wiring [26], many studies have found an apparent relationship between the MT curve and TL/L curve using more modern instrumentation. Mizusaki et al. [27] retrospectively concluded that overcorrection of the MT curve might result in less satisfactory results after STF in lumbar modifier B. This means that overcorrection of the MT curve may exacerbate the mismatch between the MT curve and TL/L curve. Ishikawa et al. [28] found that the final Cobb angle of the TL/L curve was significantly correlated with the immediate postoperative MT Cobb angle, which meant that the MT and TL/L Cobb angles matched each other. Jansen et al. [29] found a significant correlation between the relative corrections of the MT curve and the lumbar curve after STF. Similarities were noted in the present study. Comparison and correlation analyses between the postoperative MT and TL/L Cobb angle were performed. In the total group, the postoperative TL/L Cobb angle was weakly correlated with the postoperative MT Cobb angle (r: 0.350, p: 0.023), and there was no significant difference between them (p: 0.230). Going further in the subgroup analysis, in the LOSS group, the postoperative TL/L Cobb angle was not correlated with the postoperative MT Cobb angle (r: 0.074, p: 0.749), and a significant difference was found between them (p: 0.011). On the other hand, in the NO-LOSS group, the postoperative TL/L Cobb angle was moderately correlated with the postoperative MT Cobb angle (r: 0.561, p: 0.008), and no significant difference was noted between them (p: 0.420). Therefore, if the postoperative MT and TL/L Cobb angle were matched, as in the NO-LOSS group, the risk of TL/L correction loss was relatively low. If there is a mismatch between them, TL/L correction loss may occur. Nevertheless, this finding needs multicenter studies and a larger sample size for further verification.

### Limitations

First, the sample size was relatively small, but it is not easy to identify a large sample for an age- and sex-matched comparative study. A multicenter study with a larger sample may be helpful. Second, this radiographic study did not evaluate the patient’s self-assessment/satisfaction. Our next step is to explore the relationship between our findings and health-related quality of life. Third, most of the TL/L curves were moderate, and our conclusions may not be applicable to larger curves, which may not yield satisfactory outcomes after STF.

### Strengths

Our study has several major strengths. First, few studies have focused on the risk factors for TL/L correction loss following STF. This is the first study focusing on the correction loss of the unfused TL/L curve during a long-term follow-up. Second, although the relationship between MT and the TL/L curve was reported, this is the first study reporting its association with correction loss. Finally, our conclusions are meaningful for clinical practice. Good immediate postoperative spontaneous correction does not mean a satisfactory outcome at the final follow-up after STF, and close observation is needed.

## Conclusions

Posterior selective thoracic fusion is an effective treatment for AIS patients with major thoracic and secondary TL/L curves. A smaller immediate postoperative TL/L Cobb angle may be associated with TL/L correction loss during a long-term follow-up. Thus, good immediate postoperative spontaneous correction may not mean a satisfactory outcome at the final follow-up after STF. Mismatch between major thoracic and TL/L Cobb angles immediately after surgery may also be related to correction loss of the unfused TL/L curves. Although these findings were radiographic and patients were asymptomatic, close attention should be paid to smaller unfused TL/L curve and its relationship with thoracic curve in case of deterioration.

### Electronic supplementary material

Below is the link to the electronic supplementary material.

supplementary material

## Data Availability

The datasets used and/or analyzed during the current study are available from the corresponding author on reasonable request.

## List of abbreviations

AIS: Adolescent idiopathic scoliosis
3D: three-dimensional
MT: major thoracic
TL/L: thoracolumbar or lumbar
LIV: lowest instrumented vertebra
STF: selective thoracic fusion
IRB: institutional review board
AVT: apical vertebral translation
SDs: standard deviations
CI: Confidential Interval
