# Correction: Satisfactory immediate spontaneous correction may not mean satisfactory final results for moderate TL/L curves after selective thoracic fusion in AIS patients

**DOI:** 10.1186/s12891-023-06828-6

**Published:** 2023-09-05

**Authors:** Yanbin Zhang, Jing Bai, Bin Xiao, Jianguo Zhang, Da He, Yonggang Xing, Bo Liu

**Affiliations:** 1 Department of Spine Surgery, Beijing Jishuitan Hospital, Xicheng District Xinjiekou No. 31 East Street, Beijing, 100035 P.R. China; 2 Department of Trauma and Joint, The Third Affiliated Hospital of Beijing University of Traditional Chinese Medicine, Chaoyang District Anwai Xiaoguan Street No. 51, Beijing, 100029 P.R. China; 3 Department of Orthopedics of Peking Union Medical College Hospital, 1Shuai Fu Yuan, Beijing, 100730 P.R. China

**Correction:** ***BMC Musculoskelet Disord*** **24, 543 (2023)**

10.1186/s12891-023-06591-8

Following publication of the original article [1], the authors would like to delete the initial of Yonggang Xing (YGX) in the statement on the lower left quadrate of the first page of the manuscript. The line should read: YBZ and JB contributed equally and should be considered as co-first authors.

The original article [1] has been updated.
