# Supplementary material for: Satisfactory immediate spontaneous correction may not mean satisfactory final results for moderate TL/L curves after selective thoracic fusion in AIS patients
Source: BMC Musculoskelet Disord. 2023 Jul 1;24:543. doi: 10.1186/s12891-023-06591-8 (PMC10314374; doi:10.1186/s12891-023-06591-8)
Supplement: Supplementary file 1 — supplementary material [file 12891_2023_6591_MOESM1_ESM.pdf]

SPRINGER NATURE

Change of authorship request form - Journals (pre-acceptance)

Important information. Please read.

- This form should be used by authors to request any change in authorship (adding/deleting authors) including changes in corresponding authors. This form should not be used for name changes. Please fully complete all sections. Use black ink and block capitals and provide each author's full name with the given name first followed by the family name.
- By signing this declaration, all authors guarantee that the order of the authors are in accordance with their scientific contribution, if applicable as different conventions apply per discipline, and that only authors have been added who made a meaningful contribution to the work.
- Please note, in author collaborations where there is formal agreement for representing the collaboration, it is sufficient for the representative or legal guarantor (usually the corresponding author) to complete and sign the Authorship Change Form on behalf of all authors, **next to the added/removed author(s). (Complete Section 3, followed by Section 6.)**
  In author collaborations where there is no formal agreement for representing the collaboration and **there are more than 10 authors**, one may sign for all, provided the signer appends correspondence that attests that each of the authors have agreed to the change **and the added/removed authors sign the form. (Complete Section 3, followed by Section 6.)**
- Please note, we cannot investigate or mediate any authorship disputes. If you are unable to obtain agreement from all authors (including those who you wish to be removed) you must refer the matter to your institution(s) for investigation. Please inform us if you need to do this.
- If you are not able to return a fully completed form within **30 days** of the date that it was sent to the author requesting the change, we may have to withdraw your manuscript. We cannot publish manuscripts where authorship has not been agreed by all authors (including those who have been removed).
- Incomplete forms will be rejected.
- Please return/upload this form, fully completed, to the Journals Editorial Office. The Journal and/or Publisher will consider the information you have provided to decide whether to approve the proposed change in authorship. We may decide to contact your institution for more information or undertake a further investigation, if appropriate, before making a final decision.

扫描全能王 创建

SPRINGER NATURE

Change of authorship request form - Journals (pre-acceptance)

Section 1: Please provide the current title of manuscript

Manuscript ID no.: 5fe4f8c6-57c4-4d49-a3d3-4a3ac6c6b9ee

Title: Satisfactory Immediate Spontaneous Correction May Not Mean Satisfactory Final Results for Moderate TL/L Curves after Selective Thoracic Fusion in AIS

Section 2: Please provide the previous authorship, in the order shown on the manuscript before the changes were introduced. Please indicate the corresponding author by adding (CA) behind the name.

	First name(s)	Family name	ORCID or SCOPUS id, if available
1st author	Yanbin	Zhang	
2nd author	Jing	Bai	
3rd author	Bin	Xiao (CA)	
4th author	Jianguo	Zhang	
5th author	Da	He	
6th author	Yonggang	Xing	
7th author	Bo	Liu	
8th author			
9th author			
10th author			

Please use an additional sheet if there are more than 10 authors.

SPRINGER NATURE Springer Nature is one of the world's leading global research, educational and professional publishers, created in May 2015 through the combination of Nature Publishing Group, Palgrave Macmillan, Macmillan Education and Springer Science+Business Media.

扫描全能王 创建

SPRINGER NATURE

Change of authorship request form - Journals (pre-acceptance)

Section 3: Please provide a justification for change. Please use this section to explain your reasons for changing the authorship of your manuscript, e.g. what necessitated the change in authorship? Please refer to the (journal) policy pages for more information about authorship. Please explain why omitted authors were not originally included and/or why authors were removed on the submitted manuscript.

Section 4: Proposed new authorship. Please provide your new authorship list in the order you would like it to appear on the manuscript. Please indicate the corresponding author by adding (CA) behind the name. If the Corresponding Author has changed, please indicate the reason under section 3.

	First name(s)	Family name (this name will appear in full on the final publication and will be searchable in various abstract and indexing databases)	Affiliated institute	E-mail address
1st author	Yonggang	Xing	Beijing Jishuitan Hospital	dryonggangx@163.com
2nd author	Jing	Bai	The Third Affiliated Hospital of Beijing University of Traditional Chinese Medicine	359758938@163.com
3rd author	Yanbin	Zhang	Beijing Jishuitan Hospital	ybzhang_jsth@163.com
4th author	Bin	Xiao (CA)	Beijing Jishuitan Hospital	jstxiaob@163.com
5th author	Jianguo	Zhang	Peking Union Medical College Hospital	jgzhang_pumch@yahoo.com
6th author	Da	He	Beijing Jishuitan Hospital	chengbeilaoxu@163.com
7th author	Bo	Liu	Beijing Jishuitan Hospital	drliubo@sina.cn
8th author				
9th author				
10th author				

Please use an additional sheet if there are more than 10 authors.

SPRINGER NATURE Springer Nature is one of the world's leading global research, educational and professional publishers, created in May 2015 through the combination of Nature Publishing Group, Palgrave Macmillan, Macmillan Education and Springer Science+Business Media.

扫描全能王 创建

Change of authorship request form - Journals (pre-acceptance)

Section 5: Author contribution, Acknowledgement and Disclosures. Please use this section to provide a new disclosure statement and, if appropriate, acknowledge any contributors who have been removed as authors and ensure you state what contribution any new authors made (if applicable per the journal or book (series) policy). Please ensure these are updated in your manuscript - after approval of the change(s) - as our production department will not transfer the information in this form to your manuscript.

New acknowledgements:

YGX, JB and YBZ contributed equally and should be considered as co-first authors.
A special thanks to Pro. Jianguo Zhang at Peking Union Medical College Hospital.

New Disclosures (financial and non-financial interests, funding):

No special change

New Author Contributions statement (if applicable per the journal policy):

State 'Not applicable' if there are no new authors.

SPRINGER NATURE Springer Nature is one of the world's leading global research, educational and professional publishers, created in May 2015 through the combination of Nature Publishing Group, Palgrave Macmillan, Macmillan Education and Springer Science+Business Media.

扫描全能王 创建

SPRINGER NATURE

Change of authorship request form - Journals (pre-acceptance)

Section 6: Declaration of agreement. *All* authors, unchanged, new and removed *must* sign this declaration.
(NB: Please print the form, (docu)-sign and return/upload a scanned copy. Please note that signatures that have been inserted as an image file are acceptable as long as it is handwritten. Typed names in the signature box are unacceptable.) *** Please delete as appropriate. Delete all of the bold if you were on the original authorship list and are remaining as an author.**

	First name	Family name		Signature	Date
1st author	Yonggang	Xing	I agree to the proposed new authorship shown in section 4 **/and the addition/removal*of my name to the authorship list** /and the proposed change in corresponding author	Yonggang Xing	2023-6-10
2nd author	Jing	Bai	I agree to the proposed new authorship shown in section 4 **/and the addition/removal*of my name to the authorship list** /and the proposed change in corresponding author	Jin Bai	2023-6-10
3rd author	Yanbin	Zhang	I agree to the proposed new authorship shown in section 4 **/and the addition/removal*of my name to the authorship list** /and the proposed change in corresponding author	Yanbin Zhang	2023-6-10
4th authors	Bin	Xiao	I agree to the proposed new authorship shown in section 4 **/and the addition/removal*of my name to the authorship list** /and the proposed change in corresponding author	Bin Xiao	2023-6-10
5th author	Jianguo	Zhang	I agree to the proposed new authorship shown in section 4 **/and the addition/removal*of my name to the authorship list** /and the proposed change in corresponding author	Jianguo Zhang	2023-6-10
6th author	Da	He	I agree to the proposed new authorship shown in section 4 **/and the addition/removal*of my name to the authorship list** /and the proposed change in corresponding author	Da HE	2023-6-10
7th author	Bo	Liu	I agree to the proposed new authorship shown in section 4 **/and the addition/removal*of my name to the authorship list** /and the proposed change in corresponding author	Bo Liu	2023-6-10

SPRINGER NATURE Springer Nature is one of the world's leading global research, educational and professional publishers, created in May 2015 through the combination of Nature Publishing Group, Palgrave Macmillan, Macmillan Education and Springer Science+Business Media.

扫描全能王 创建

Change of authorship request form - Journals (pre-acceptance)

	First name	Family name		Signature	Date
8th author			I agree to the proposed new authorship shown in section 4 **/and the addition/removal*of my name to the authorship list** /and the proposed change in corresponding author		
9th author			I agree to the proposed new authorship shown in section 4 **/and the addition/removal*of my name to the authorship list** /and the proposed change in corresponding author		
10th author			I agree to the proposed new authorship shown in section 4 **/and the addition/removal*of my name to the authorship list** /and the proposed change in corresponding author		

Please use an additional sheet if there are more than 10 authors.

In case of author collaborations with formal agreement:

	Name of consortium/consortia	First name	Family name		Signature	Date
Representative/legal guarantor				I agree to the proposed new authorship shown in section 4 **/and the addition/removal*of my name to the authorship list** /and the proposed change in corresponding author		

Both added/removed authors should complete the information in the first table under Section 6.

---- End of form ----

扫描全能王 创建
